# Supplementary material for: Reduction in diarrhea cases following implementation of COVID-19 hand hygiene interventions in Ghana: A causal impact analysis
Source: PLoS One. 2024 Aug 29;19(8):e0309202. doi: 10.1371/journal.pone.0309202 (PMC11361678; doi:10.1371/journal.pone.0309202)
Supplement: Attachment — Submitted filename: Point by point rebuttal_on reviewers comments_final.docx [file pone.0309202.s003.docx]

**Response to Reviewers**

**Point by point rebuttal on reviewers’ comments**

**Journal Requirements:**

1. When submitting your revision, we need you to address these additional requirements.

Please ensure that your manuscript meets PLOS ONE's style requirements, including those for file naming. The PLOS ONE style templates can be found at 
https://journals.plos.org/plosone/s/file?id=wjVg/PLOSOne_formatting_sample_main_body.pdf and 
<https://journals.plos.org/plosone/s/file?id=ba62/PLOSOne_formatting_sample_title_authors_affiliations.pdf>

**Response:** Title has now been revised to align with this requirement and also addresses review comments which suggested we replace the word “Impact” with another which does not suggest causality.

The title now reads **“Reduction in Diarrhea Cases Following Implementation of COVID-19 Hand Hygiene Interventions in Ghana: A Causal Impact Analysis”**

2. You indicated that ethical approval was not necessary for your study. We understand that the framework for ethical oversight requirements for studies of this type may differ depending on the setting and we would appreciate some further clarification regarding your research. Could you please provide further details on why your study is exempt from the need for approval and confirmation from your institutional review board or research ethics committee (e.g., in the form of a letter or email correspondence) that ethics review was not necessary for this study? Please include a copy of the correspondence as an ""Other"" file.

**Response**: This study utilized only aggregated secondary data and was carried out as part of efforts to improve quality of both COVID-19 and diarrhea diseases management in Ghana. This study aligns with Helsinki’s ethical guidelines on the use of secondary data and did not require ethical approval since the study only used aggregated secondary data which did not contain personal identifiers of patients.

DHIMS 2 primarily collects and reports aggregated health data rather than individual-level health data significantly reducing the risk of identification or harming individual patients. Many ethical guidelines and regulations, including those from the World Health Organization (WHO) and national ethics committees, provide exemptions for research using aggregated or anonymized data, recognizing minimal risks to individuals when data cannot be linked to specific persons.

The authors have also listed below three papers published in PLOS One which used DHIMS 2 data and did not require ethical clearance.

References

1.	Malm KL, Peprah NY, Mohammed W, Adomako BY, Oppong S, Boateng P, et al. A retrospective analysis of malaria deaths in the pre- and intra- COVID 19 pandemic era, Ghana, 2016–2021. PLoS One [Internet]. 2024;19(2 February):2016–21. Available from: http://dx.doi.org/10.1371/journal.pone.0286212

2.	Kayode GA, Amoakoh-Coleman M, Brown-Davies C, Grobbee DE, Agyepong IA, Ansah E, et al. Quantifying the validity of routine neonatal healthcare data in the Greater Accra Region, Ghana. PLoS One. 2014;9(8).

3.	Awine T, Malm K, Peprah NY, Silal SP. Spatio-temporal heterogeneity of malaria morbidity in Ghana: Analysis of routine health facility data. PLoS One. 2018;13(1).

3. In the online submission form, you indicated that The data underlying the results presented in the study are available from the corresponding author upon reasonable request.
 
All PLOS journals now require all data underlying the findings described in their manuscript to be freely available to other researchers, either a. In a public repository, b. Within the manuscript itself, or c. Uploaded as supplementary information.
This policy applies to all data except where public deposition would breach compliance with the protocol approved by your research ethics board. If your data cannot be made publicly available for ethical or legal reasons (e.g., public availability would compromise patient privacy), please explain your reasons on resubmission and your exemption request will be escalated for approval.

**Response**: The data used in the analysis for this paper has been uploaded as a supplementary information.

4. We note that Figure 1 in your submission contain map images which may be copyrighted. All PLOS content is published under the Creative Commons Attribution License (CC BY 4.0), which means that the manuscript, images, and Supporting Information files will be freely available online, and any third party is permitted to access, download, copy, distribute, and use these materials in any way, even commercially, with proper attribution. For these reasons, we cannot publish previously copyrighted maps or satellite images created using proprietary data, such as Google software (Google Maps, Street View, and Earth). For more information, see our copyright guidelines: http://journals.plos.org/plosone/s/licenses-and-copyright.
 
We require you to either present written permission from the copyright holder to publish these figures specifically under the CC BY 4.0 license, or remove the figures from your submission:
 
a. You may seek permission from the original copyright holder of Figure 1 to publish the content specifically under the CC BY 4.0 license.  
 
We recommend that you contact the original copyright holder with the Content Permission Form (http://journals.plos.org/plosone/s/file?id=7c09/content-permission-form.pdf) and the following text:
“I request permission for the open-access journal PLOS ONE to publish XXX under the Creative Commons Attribution License (CCAL) CC BY 4.0 (http://creativecommons.org/licenses/by/4.0/). Please be aware that this license allows unrestricted use and distribution, even commercially, by third parties. Please reply and provide explicit written permission to publish XXX under a CC BY license and complete the attached form.”
 
Please upload the completed Content Permission Form or other proof of granted permissions as an "Other" file with your submission.
 
In the figure caption of the copyrighted figure, please include the following text: “Reprinted from [ref] under a CC BY license, with permission from [name of publisher], original copyright [original copyright year].”
 
b. If you are unable to obtain permission from the original copyright holder to publish these figures under the CC BY 4.0 license or if the copyright holder’s requirements are incompatible with the CC BY 4.0 license, please either i) remove the figure or ii) supply a replacement figure that complies with the CC BY 4.0 license. Please check copyright information on all replacement figures and update the figure caption with source information. If applicable, please specify in the figure caption text when a figure is similar but not identical to the original image and is therefore for illustrative purposes only.
The following resources for replacing copyrighted map figures may be helpful:
 
USGS National Map Viewer (public domain): http://viewer.nationalmap.gov/viewer/
The Gateway to Astronaut Photography of Earth (public domain): http://eol.jsc.nasa.gov/sseop/clickmap/
Maps at the CIA (public domain): https://www.cia.gov/library/publications/the-world-factbook/index.html and https://www.cia.gov/library/publications/cia-maps-publications/index.html
NASA Earth Observatory (public domain): http://earthobservatory.nasa.gov/
Landsat: http://landsat.visibleearth.nasa.gov/
USGS EROS (Earth Resources Observatory and Science (EROS) Center) (public domain): http://eros.usgs.gov/#
Natural Earth (public domain): <http://www.naturalearthdata.com/>

**Response: New Figure 1 has been uploaded which was constructed by authors.**

5. Please review your reference list to ensure that it is complete and correct. If you have cited papers that have been retracted, please include the rationale for doing so in the manuscript text, or remove these references and replace them with relevant current references. Any changes to the reference list should be mentioned in the rebuttal letter that accompanies your revised manuscript. If you need to cite a retracted article, indicate the article’s retracted status in the References list and also include a citation and full reference for the retraction notice.

**Response: All references have been updated to PLOS ONE standards**.

[Note: HTML markup is below. Please do not edit.]

Reviewers' comments:

Reviewer's Responses to Questions

**Comments to the Author**

1. Is the manuscript technically sound, and do the data support the conclusions?

The manuscript must describe a technically sound piece of scientific research with data that supports the conclusions. Experiments must have been conducted rigorously, with appropriate controls, replication, and sample sizes. The conclusions must be drawn appropriately based on the data presented.

Reviewer #1: Partly

Reviewer #2: Yes

2. Has the statistical analysis been performed appropriately and rigorously?

Reviewer #1: I Don't Know

Reviewer #2: Yes

3. Have the authors made all data underlying the findings in their manuscript fully available?

The [PLOS Data policy](http://www.plosone.org/static/policies.action#sharing) requires authors to make all data underlying the findings described in their manuscript fully available without restriction, with rare exception (please refer to the Data Availability Statement in the manuscript PDF file). The data should be provided as part of the manuscript or its supporting information, or deposited to a public repository. For example, in addition to summary statistics, the data points behind means, medians and variance measures should be available. If there are restrictions on publicly sharing data—e.g. participant privacy or use of data from a third party—those must be specified.

Reviewer #1: No

Reviewer #2: Yes

4. Is the manuscript presented in an intelligible fashion and written in standard English?

PLOS ONE does not copyedit accepted manuscripts, so the language in submitted articles must be clear, correct, and unambiguous. Any typographical or grammatical errors should be corrected at revision, so please note any specific errors here.

Reviewer #1: Yes

Reviewer #2: Yes

5. Review Comments to the Author

Please use the space provided to explain your answers to the questions above. You may also include additional comments for the author, including concerns about dual publication, research ethics, or publication ethics. (Please upload your review as an attachment if it exceeds 20,000 characters)

**Reviewer #1:** GENERAL REMARKS
The paper pinpoints the contribution of hand washing introduced during the COVID-19 to a decrease in reported diarrhea cases in Ghana. The paper is generally well written and flows very well.
The key concern about the paper is the use of the word “impact” as this suggests causality. The authors use the word impact based on the statistical package used. Even though the statistical package may have this property, I’m not sure there is guarantee that all factors that could have contributed to the reduction in diarrhea cases were accounted for. This is especially considering that that in the sub-national analyses, not all the regions showed a significant reduction in diarrhea cases.
It is suggested that “association” is used instead of the word “impact” in the paper.
The authors also need to provide references for some statements made and these areas are highlighted in the paper.
TITLE
For reasons given above it is suggested that “association” is used instead of the word “impact” which suggests causality but in my view is not proven beyond doubt.

**Response:** Thanks for the comment and suggestion. The title now reads **“Reduction in Diarrhea Cases Following Implementation of COVID-19 Hand Hygiene Interventions in Ghana: A Causal Impact Analysis”**

INTRODUCTION
The introduction is well written but the same comment applies to the use of the word “impact on page 3 Line 89.

**Response**: Thanks for this comment. “Effects” has been used in place of “Impact”. This can be found on line 96 in the main manuscript.

METHODS
Lines 140 on page 5. Please provide more information on the incorporated covariates
**Response:** Out-patient department (OPD) attendance was included as a covariate because it reflects health-seeking behavior which can influence the number of diarrheal cases recorded. Including the out-patient department attendance as a covariate would account for changes in diarrhea cases that might result from other interventions.

Statistical Analysis
In reference to Lines 133 to 138 on page 5.
The likelihood is that some readers may not fully understand this narrative (especially the term counterfactual scenario) or may not be familiar with the Causal Impact package.
I suggest that this section on the statistical analyses is simplified to enable readers really appreciate the point that it was indeed the introduction of the hand washing that caused the decrease in diarrhea cases. Better still I propose association instead of impact because there is no guarantee all factors were accounted for.

**Response:** This section of the manuscript has been rewritten to explain the term “Counterfactual” [what would have happened if the Covid-19 hand hygiene hand not been implemented] to the understanding of the reader. This can be found on lines 148 to 157. Furthermore, a reference on causal impact package has been added. This is reference number 26 in the main manuscript.

Lines 141-142 Please provide information on the OPD data and explain how the OPD attendance was controlled for.

**Response:** Data on OPD attendance was collected over the period pre-and-post implementation of the hand hygiene protocol. This served as a baseline to account for potential changes in health-seeking behavior unrelated to the introduction of the hand hygiene protocol. By including the number of OPD attendance as a covariate in the model, the model adjusted for any variation in the number of patients making OPD visit. This helped isolate the specific impact of the hand hygiene protocol on diarrhea cases, ensuring that the observed effects were not confounded by changes in OPD attendance.

RESULTS
Lines 149, 156 Please provide references

Response: Please references have been provided in the main manuscript as reference 27 (found on line 169) and reference 28 (found on line 174).

Lines 185 to 204 Please present data in a table
**Response**: Data is presented in a table as suggested by reviewer. This is presented as Table 2 in the main manuscript as found on line 213.


DISCUSSION
1.In reference to these statements:
It is crucial to remember, nevertheless, that not all locations saw statistically significant
decreases. Some regions, such as Bono East, North-East, Oti, and Western North, demonstrated
non-significant changes in diarrheal cases.
.
On page 19 in the Discussion, an argument was made that Greater Accra and Ashanti showed higher reductions in diarrhea cases and were the hardest hit.
Following that argument please comment on whether these regions that didn't show significant reductions were the least hit.

**Response**: During the COVID-19 pandemic, the Greater Accra region was termed the “hotspot” area which led to the implementation of most of the COVID-19 restrictions such as lockdowns, restriction on public gatherings, the closure of borders in these regions for public safety and the institution of mandatory hand hygiene protocols at all public places (References 36,37) found on lines 274 to 279 in the main manuscript.

2. In reference to the following statement:
These disparities might stem from variations in socio-economic factors, healthcare infrastructure, cultural practices, or other unmeasured contextual variables impacting the efficacy of the hand hygiene protocol in these areas.

What is the evidence to suggest this? Please back it up with evidence from literature and references.

**Response**: This may be because these are newly created regions from the already existing 10 regions in Ghana. These newly created regions are now grappling with basic amenities and infrastructure like roads, good healthcare, electricity and access to potable water and improved sanitation.


CONCLUSION
In line with this first statement of the conclusion
The findings suggest an association between the implementation of COVID-19 handwashing
protocols and reduced diarrheal disease burden in Ghana.

The suggestion is to use "associated" instead of "impact" in the paper since causality (hand washing causing a degree in diarrhea cases) cannot be 100% proven as all possible factors were not controlled for.

**Response:** The word impact has been changed to “association” as found on line 314 in main manuscript.

**Reviewer #2:** General Comment
Overall, the topic is interesting and the manuscript is well-written. The main results highlight the reduction in diarrheal cases in Ghana due to the COVID-19 hand hygiene protocol, using observed data from the pre-protocol and protocol periods as well as a counterfactual model in the "causal impact R package (Bayesian Structural Time Series Model)." Please check the entire manuscript for consistency and to avoid typos.

Specific Comments and Suggestions
1.In the “Statistical Analysis” section under “METHODS,” the author mentioned that a two-sample t-test was conducted. Were any other statistical tests, in addition to the two-sample t-test, applied? The Mann-Whitney test was observed in some figures comparing summary measures between the pre-protocol and protocol periods (Figure 2-A to D).

**Response:** Assumptions underlying two-sample t-test such as normality of the outcome variable (diarrhoea cases) and equality of variance in each group were tested, and where the outcome was not normally distributed between groups (Pre-and-Post protocol) a non-parametric Mann-Whitney test was conducted, and where variances were not equal among groups a two-sample t-test with unequal variance were estimated. This can be found on lines 143 to 148.

2.The author mentioned that OPD attendance was considered a potential confounder and controlled for (line number 141), but in the “DISCUSSION” section, the fact that potential confounding variables were not considered throughout the study is reported as one of the limitations.

Response: This error has been addressed. OPD attendance was used as the potential confounding variable in this study. This can be found on lines 308 to 310 in the main manuscript.

3.In Table 1 of the “RESULTS” section, please recheck the numbers in the “Sum” column, as the addition of diarrheal cases during the pre-protocol and protocol periods slightly differs from the total cases in Ghana.

**Response:** The error in the summation of the number of diarrhea cases presented in table 1 may be due to typo error. This has been corrected to reflect the true summation of the number of diarrhea cases pre-and-post intervention. This can be found on line 195 in the main manuscript.

4.In line numbers 176 and 207, the figure numbers referenced by the author are incorrect (Figure 1 was incorrectly mentioned instead of Figure 2, as Figure 1 refers to the map of Ghana).

**Response:** The figures have been appropriately referenced in the current version of the manuscript for resubmission.

5.When presenting the results for the “Sub-national analysis of the difference in diarrhea cases prior to and during the introduction of the COVID-19 handwashing protocol in Ghana,” I suggest presenting these results in a table (similar to Table 2 for presenting causal impact) for better readability and clarity. A table will not duplicate the figures (Figure 2, 2A to 2D) because it will include the mean difference with 95% CI, t-statistics, p-value, and Cohen’s d with 95% CI, which are not presented in the current figures.

**Response:** The graphs/plots have been converted into tables as suggested by the reviewer to reflect the relevant statistics mentioned by the reviewer. This can be found in Table 2, line 213 in the main manuscript.

6.I suggest conducting a subgroup analysis by age strata if data is available. (In DHIMS 2, if the age-group variable of the aggregate data for diarrheal cases is available, the results for the reduction in diarrheal cases for specific age groups, such as under-five, would be more interesting.)

**Response:** This is an excellent suggestion from the reviewer, however, data on the number of cases of diarrhea reported in health facilities that are entered in to DHIMS 2 are figures aggregated on monthly basis, and are not linked to patients, therefore age-specific analysis would not be possible.

6. PLOS authors have the option to publish the peer review history of their article ([what does this mean?](https://journals.plos.org/plosone/s/editorial-and-peer-review-process#loc-peer-review-history)). If published, this will include your full peer review and any attached files.


If you choose “no”, your identity will remain anonymous but your review may still be made public.


**Do you want your identity to be public for this peer review?** For information about this choice, including consent withdrawal, please see our [Privacy Policy](https://www.plos.org/privacy-policy).

Reviewer #1: No

Reviewer #2: No
